# Detecting missing IS-A relations in the NCI Thesaurus using an enhanced hybrid approach

**DOI:** 10.1186/s12911-020-01289-6

**Published:** 2020-12-15

**Authors:** Fengbo Zheng, Rashmie Abeysinghe, Nicholas Sioutos, Lori Whiteman, Lyubov Remennik, Licong Cui

**Affiliations:** 1 Department of Computer Science, University of Kentucky, Lexington, KY USA; 2 Department of Neurology, McGovern School of Medicine, University of Texas Health Science Center at Houston, Houston, TX USA; 3 Enterprise Vocabulary Services, Center for Biomedical Informatics and Information Technology, National Cancer Institute, Bethesda, MD USA; 4 School of Biomedical Informatics, University of Texas Health Science Center at Houston, Houston, TX USA

**Keywords:** Quality assurance, NCI Thesaurus, Role definition, Lexical feature

## Abstract

**Background:**

The National Cancer Institute (NCI) Thesaurus provides reference terminology for NCI and other systems. Previously, we proposed a hybrid prototype utilizing lexical features and role definitions of concepts in non-lattice subgraphs to identify missing IS-A relations in the NCI Thesaurus. However, no domain expert evaluation was provided in our previous work. In this paper, we further enhance the hybrid approach by leveraging a novel lexical feature—roots of noun chunks within concept names. Formal evaluation of our enhanced approach is also performed.

**Method:**

We first compute all the non-lattice subgraphs in the NCI Thesaurus. We model each concept using its role definitions, words and roots of noun chunks within its concept name and its ancestor’s names. Then we perform subsumption testing for candidate concept pairs in the non-lattice subgraphs to automatically detect potentially missing IS-A relations. Domain experts evaluated the validity of these relations.

**Results:**

We applied our approach to 19.08d version of the NCI Thesaurus. A total of 55 potentially missing IS-A relations were identified by our approach and reviewed by domain experts. 29 out of 55 were confirmed as valid by domain experts and have been incorporated in the newer versions of the NCI Thesaurus. 7 out of 55 further revealed incorrect existing IS-A relations in the NCI Thesaurus.

**Conclusions:**

The results showed that our hybrid approach by leveraging lexical features and role definitions is effective in identifying potentially missing IS-A relations in the NCI Thesaurus.

## Background

The National Cancer Institute (NCI) Thesaurus covers knowledge in a wide range of cancer research domains including disease, findings and abnormalities; agents, drugs and chemicals; genes and gene products, etc. [1, 2] It facilitates data sharing and interoperability across different NCI systems [3, 4]. It has also been widely used for coding, knowledge reference and public reporting by other partners such as U.S. Food and Drug Administration [5, 6].

Given the important roles that the NCI Thesaurus plays, its quality issues such as missing IS-A relations, if not addressed, will affect the quality of its downstream systems or applications. For example, suppose we are using NCI Thesaurus-based search engines to identify a cohort of patients with “Cystic Neoplasm”. The engines will look for patients with diseases that are descendants of “Cystic Neoplasm”. However, “Dermoid Cyst” is currently not listed as one of its descendants (i.e., a missing IS-A relation) in the NCI Thesaurus. As a consequence, patients with “Dermoid Cyst” will be missing from the cohort search result.

To identify missing IS-A relations in the NCI Thesaurus, in our previous work [7], we proposed a hybrid model in which role definitions of concepts in non-lattice subgraphs are harmonized with lexical features to assist with the subsumption checking of IS-A relations between concept pairs. Although a preliminary evaluation based on different versions of the NCI Thesaurus showed that the hybrid model is promising, no domain expert evaluation was performed to assess the effectiveness of our hybrid model. In this paper, we enhance our previous work and introduce roots of noun chunks within concept names as a new lexical feature to minimize invalid identification of missing IS-A relations. Here a noun chunk is a noun plus the words describing the noun, and the noun is considered as the root of the noun chunk. To evaluate the effectiveness of our enhanced approach, we provide the NCI Enterprise Vocabulary Services (EVS) domain experts with the potentially missing IS-A relations identified. A comparison between this enhanced approach and our previous lexical-based approach is also provided to show the effectiveness of leveraging both lexical features and role definitions.

### Related work on identifying missing IS-A relations

Given its importance, auditing completeness of hierarchical IS-A relations has been an active research area. Ochs et al. proposed abstraction networks to identify areas that contain quality issues including missing IS-A relations [8]. Chen et al. split Unified Medical Language System (UMLS) concepts into semantic-uniform sets and recursively added small set into larger set in order to detect missing hierarchical relations [9]. Bodenreider identified missing IS-A relations in the SNOMED CT by considering lexical features (i.e., words in concept names) of concepts as their logical definitions [10]. Quesada-Martínez et al. analyzed concept names in the SNOMED CT to identify lexical regularities and suggested missing relations [11]. Abeysinghe et al. introduced a lexical-based inference approach to derive hierarchical inconsistencies and uncover missing IS-A relations in the SNOMED CT, NCI Thesaurus and Gene Ontology [12]. Liu et al. created embeddings for each concept based on its related IS-A relations and used convolutional neural network to discover missing IS-A relations between neoplasm concepts in the NCI Thesaurus [13]. In our previous works [14–20], we found that non-lattice subgraphs often reveal quality issues including missing IS-A relations. For instance, lexical-based approaches based on non-lattice subgraphs were developed to identify missing IS-A relations in the SNOMED CT [17, 18] and NCI Thesaurus [19, 20]. Recently, Sun et al. used convolutional neural network combined with multilayer perception classifier to assist with the validation of missing IS-A relations in non-lattice subgraphs of the SNOMED CT [21].

### Lexical features and role definitions in biomedical ontologies

To automatically identify missing IS-A relations, one commonly used approach is to find features to represent the meanings of concepts [10, 13, 17–21] and check whether there exists any subsumption relation between the represented meanings. In biomedical ontologies, two important aspects can be utilized to represent the semantic meaning of a concept—lexical features and role definitions.

Lexical features (e.g., words appearing in concept names) have been widely adopted to detect missing hierarchical relations in ontologies including the NCI Thesaurus, Gene Ontology and SNOMED CT. However, in many cases, it is challenging to get the machine to catch the meanings and other details behind the words. Take concept “Sarcoma” in the NCI Thesaurus as an example. Purely from the concept name itself, the machine will not be able to know that this concept refers to a malignant neoplasm of the soft tissue or bone. In addition, concept names are defined manually by curators of biomedical ontologies and inconsistencies may exist during the naming process [22], which may further affect the subsumption checking.

When it comes to role definitions, most modern ontologies provide formally defined role definitions (in a format of relations between concepts) to represent meanings of concepts. There are two types of relations involved in the role definition of a concept: hierarchical IS-A relations and associative roles, where the IS-A relations determine the concept’s location in the hierarchy (e.g., its supertypes and its subtypes) and the associative roles are the role assertions that further define the concept. We denote the role definition of a concept as (*role*, *value*) pairs, where *value* refers to the target concept to which this concept connects through the *role*. Table 1 shows role definitions of concept “Sarcoma” in the NCI Thesaurus consisting of two IS-A relations and 11 associative roles. For example, (*IS*-*A*, $Malignant\ Neoplasm$) is an IS-A relation while ($Disease\_Has\_Associated\_Anatomic\_Site$, $Connective\ and\ Soft\ Tissue$) is an associative role. These kinds of role definitions refine the meanings of concepts. However, role definitions are often incomplete making them impractical to be solely used in representing meanings of concepts. For instance, in 19.08d version of the NCI Thesaurus, only 17,052 out of 146,688 (11.62%) concepts are considered as fully defined in role definition; and in 11/02/2019 release of the Gene Ontology, the number is 12,011 out of 44,650 (26.9%).

**Table 1:** The role definition of concept “Sarcoma” (C9118) in NCI Thesaurus

Role	Value
IS-A	Connective and soft tissue neoplasm
IS-A	Malignant neoplasm
Disease_Has_Abnormal_Cell	Malignant cell
Disease_Has_Abnormal_Cell	Neoplastic cell
Disease_Excludes_Normal_Cell_Origin	Epithelial cell
Disease_Excludes_Normal_Tissue_Origin	Epithelial tissue
Disease_Has_Associated_Anatomic_Site	Connective and soft tissue
Disease_Has_Normal_Tissue_Origin	Connective and soft tissue
Disease_Excludes_Finding	Benign cellular infiltrate
Disease_Excludes_Finding	Indolent clinical course
Disease_Excludes_Finding	Intermediate filaments present
Disease_Excludes_Finding	Intracytoplasmic eosinophilic inclusion
Disease_Has_Finding	Malignant cellular infiltrate

In this paper, in order to derive more precise missing IS-A relations from subsumption testing, we further enhance our hybrid model by harmonizing lexical features and role definitions to represent the meanings of concepts.

## Methods

In our previous works [16–19], we found that non-lattice subgraphs in biomedical ontologies often reveal quality issues including missing IS-A relations. Therefore, in this work, we focus on non-lattice subgraphs to detect missing IS-A relations. To identify missing IS-A relations in non-lattice subgraphs, we first find a proper way to represent the meanings of concepts, and then check whether there exist any subsumption relations between the represented meanings of unlinked concepts (i.e., not connected by IS-A relations either directly or transitively) within non-lattice subgraphs.

There are mainly three steps: (1) compute non-lattice subgraphs and identify candidate pairs of concepts, which are currently not linked by IS-A relations; (2) model each concept by harmonizing the role definition, words and roots of noun chunks within its concept name and its ancestor’s names, to represent its meanings; (3) perform subsumption checking for candidate pairs based on our hybrid model.

### Computing non-lattice subgraphs and generating candidate pairs

Concepts in an ontology are organized into an IS-A hierarchy, which can be considered as a directed acyclic graph. Given two concepts *A* and *B* in the ontology, a common ancestor *X* of *A* and *B* is known as their *minimal* common ancestor, if *A* and *B* do not have any other common ancestor *Y* such that *X* is an ancestor of *Y*. Similarly, a common descendant *P* of *A* and *B* is known as their *maximal* common descendant, if *A* and *B* do not have any other common descendant *Q* such that *P* is a descendant of *Q*. An ontology forms a *lattice* if any two concepts in the ontology have a unique minimal common ancestor and a unique maximal common descendant. Lattice is a desirable property for a well-formed ontology or terminology [14].

**Fig. 1:**
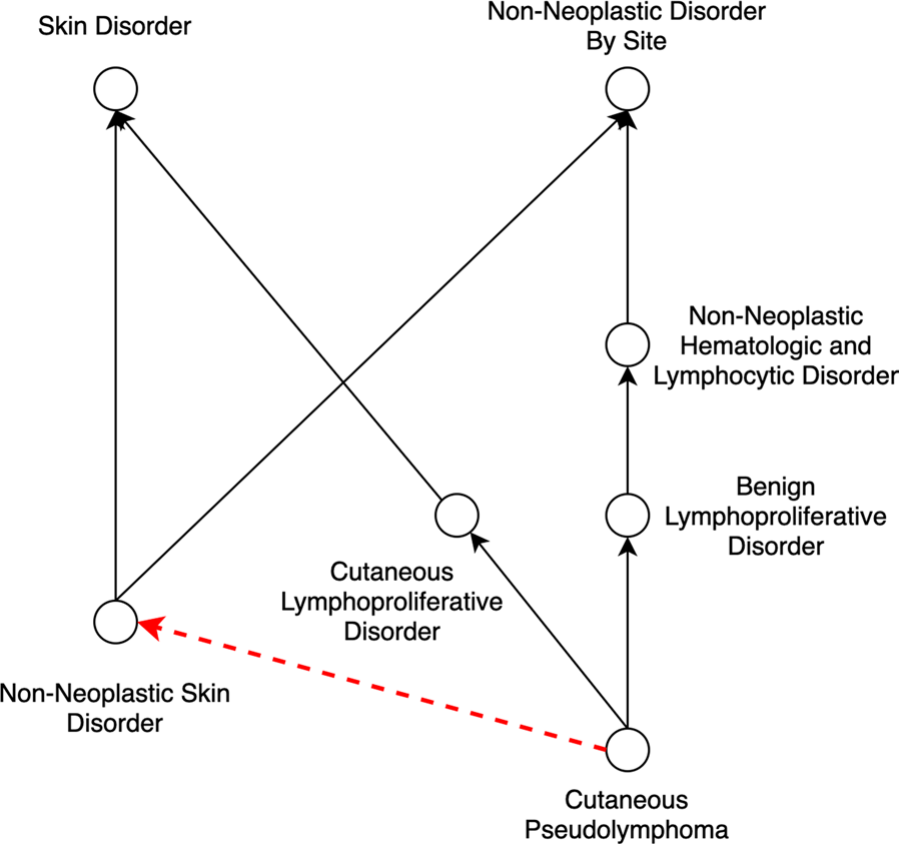
An example of non-lattice subgraphs in 19.08d version of NCI Thesaurus. Concepts are connected by IS-A relations. The red dotted line shows a potentially missing IS-A relation between concepts “Cutaneous Pseudolymphoma” and “Non-Neoplastic Skin Disorder” identified by our method

A pair of concepts is called as a *non-lattice pair* if the two concepts have more than one maximal common descendant. A *non-lattice subgraph* can be obtained from a non-lattice pair by first reversely computing the minimal common ancestors of the maximal common descendants of the non-lattice pair; and then aggregating all the concepts and IS-A relations between them [17]. Figure 1 shows a non-lattice subgraph in the NCI Thesaurus (19.08d version) obtained from the non-lattice pair (“Skin Disorder”, “Non-Neoplastic Disorder By Site”) with two maximal common descendants “Non-Neoplastic Skin Disorder” and “Cutaneous Pseudolymphoma”.

We leverage an efficient non-lattice extraction algorithm [23] to compute all the non-lattice subgraphs in the NCI Thesaurus. Then we identify potentially missing IS-A relations between pairs of concepts (denoted as candidate pairs), which are currently not linked by IS-A relations in the non-lattice subgraphs. Take the non-lattice subgraph shown in Fig. 1 as an example, (“Non-Neoplastic Skin Disorder”, “Cutaneous Pseudolymphoma”) is a candidate pair and (“Skin Disorder”, “Benign Lymphoproliferative Disorder”) is another.

**Fig. 2:**
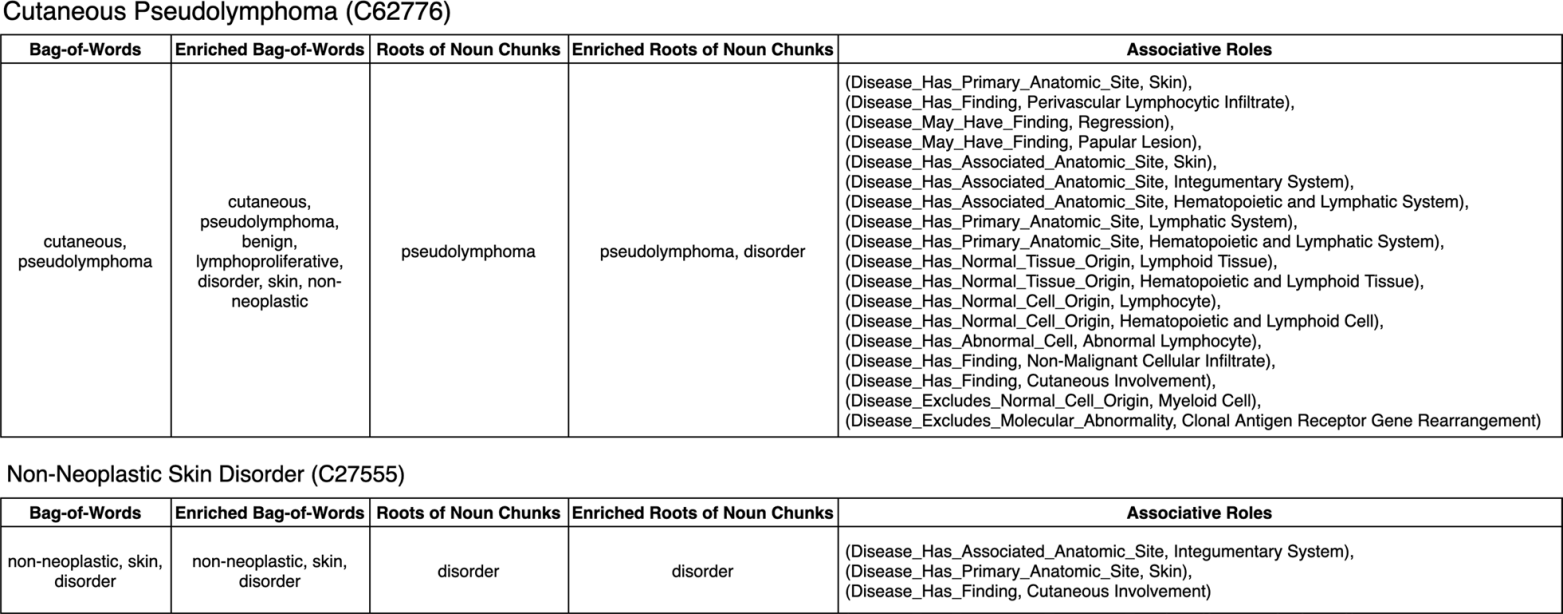
Semantic models of concepts “Cutaneous Pseudolymphoma (C62776)” and “Non-Neoplastic Skin Disorder (C27555)” that are contained in non-lattice subgraph shown in Fig. 1

### Modeling concepts

In this work, we introduce a comprehensive semantic model that utilizes role definitions and lexical features to represent the meanings of concepts.

Given a concept *C*, its semantic model contains five parts ($C_{bow}$, $C_{ebow}$, $C_r$, $C_{er}$, $C_a$):

1. bag-of-words $C_{bow}$, which includes words appearing in its preferred name;
2. enriched bag-of-words $C_{ebow}$, which includes words appearing in its preferred name and words in its ancestors’ preferred names;
3. roots of noun chunks $C_r$, which includes roots of noun chunks in its preferred name;
4. enriched roots of noun chunks $C_{er}$, which includes roots of noun chunks in its preferred name and in its ancestors’ preferred names; and
5. associative roles $$C_a$$.

Figure 2 shows the semantic models for concepts “Cutaneous Pseudolymphoma” and “Non-Neoplastic Skin Disorder” in the non-lattice subgraph shown in Fig. 1. Note that IS-A relations in the role definitions are not included in the semantic model, since our goal is to identify missing IS-A relations. Alternatively, we use features inherited from the concept’s ancestor (i.e., $C_{ebow}$ and $C_{er}$) to embody the IS-A relations, which can gather more concept related information and thus help refine the meaning of the concept. We maintain both original lexical features (i.e., $C_{bow}$ and $C_r$) and enriched ones (i.e., $C_{ebow}$ and $C_{er}$) for performing subsumption testing later.

#### Lexical features

The regular bag-of-words $C_{bow}$ and enriched bag-of-words $C_{ebow}$ can convey the meaning of a concept to some extent. However, there exist some words that may express different meanings depending on the contexts under which they appear. For example, word “erlotinib” in concept “Erlotinib” and in concept “Erlotinib Hydrochloride” convey different meanings—the former refers to the chemical item itself while the later is used to describe word “hydrochloride”. Therefore, even though both concepts contain the same word “erlotinib”, it should be considered as a different lexical feature for each concept.

To handle such cases (i.e., noun used as a descriptive term), our idea is to leverage a technique in Natural Language Processing (NLP) called dependency parsing, which can extract the grammatical structure and relationships between words for a given phrase. For example, after parsing concept name “Malignant Bladder Neoplasm”, we can get “malignant bladder neoplasm” whole as a noun chunk. The word “malignant” is used to modify “neoplasm” in terms of the type (i.e., benign or malignant) while the word “bladder” is used to modify “neoplasm” in term of the location (i.e., anatomic site). In this work, besides bag-of-words $C_{bow}$ (and enriched $C_{ebow}$), we also adopt roots of noun chunks $C_r$ (and enriched $C_{er}$) as part of the lexical feature. Given a concept name, we use spaCy [24], an open-source library for NLP, to parse it and recognize the roots of noun chunks. In the previous example, “neoplasm” is denoted as a root of noun chunk since other words are used to modify it. By utilizing roots of noun chunks $C_r$, to some extent we can distinguish different meanings of a word in different context. In the concepts “Erlotinib” and “Erlotinib Hydrochloride”, word “erlotinib” will be taken as two different words—a root of noun chunk in the former concept, but a descriptive term (i.e., not a root of noun chunk) in the latter concept.

In this work, we also adopt a list of stop words that may distort the represented meanings of concepts. As mentioned in our previous work [7], concept names that contain “and” are often inconsistent with what they actually mean and their role definitions. For example, concept “Lip and Oral Cavity Squamous Cell Carcinoma” actually refers to a squamous cell carcinoma arising from the lip or the oral cavity. In this work, we do not perform subsumption testing for candidate pairs that include concepts whose $C_{bow}$ contain any stop word. In addition, while generating enriched lexical features $C_{ebow}$ and $C_{er}$, concepts will not inherit lexical features $C_{bow}$ and $C_r$ from their ancestors containing any stop word such that the stop words will not propagate. More specifically, as long as an ancestor contains a stop word, none of the ancestor’s lexical features will be inherited. The list of stop words used in this step is the same as the one used in our previous lexical-based method [20].

In Fig. 2, it can be seen that concept “Cutaneous Pseudolymphoma” has two single words and inherits seven words from its ancestors such as “Benign Lymphoproliferative Disorder”, “Skin Disorder” and “Non-Neoplastic Disorder”, which enrich the meaning expressed by the concept. Also, word “pseudolymphoma” is recognized as the root of noun chunk “cutaneous pseudolymphoma”. Concept “Cutaneous Pseudolymphoma” also inherits another root of noun chunk “disorder” from its ancestor “Benign Lymphoproliferative Disorder”. Note that another ancestor of concept “Cutaneous Pseudolymphoma” is “Non-Neoplastic Hematologic and Lymphocytic Disorder”, which contains the stop word “and”. Hence, $C_{bow}$ and $C_r$ of this ancestor are not inherited.

#### Associative roles

In our model, we use associative roles $C_a$ to collect and adjust the meaning of concepts which may not be fully expressed by lexical features, especially for concepts that are lexically similar but should not be linked by IS-A relations. Consider the concepts “Metastatic Malignant Neoplasm in the Pancreas” and “Metastatic Malignant Pancreatic Neoplasm”. If only lexical features are considered, the former concept’s lexical features include all of the latter one’s after the enrichment (e.g., “Metastatic Malignant Neoplasm in the Pancreas” inherits “pancreatic” from its ancestor “Pancreatic Neoplasm”). However, the former concept “Metastatic Malignant Neoplasm in the Pancreas” refers to a malignant neoplasm that has spread to the pancreas from another anatomic site, while “Metastatic Malignant Pancreatic Neoplasm” actually refers to a malignant neoplasm that arises from the pancreas and has metastasized to another anatomic site. Thus, there should not be any subsumption relation between these two concepts. However, the difference between the two concepts can not be caught purely from their lexical features. To compensate this, we adopt associative roles, which usually contain information that is not included in the literal meanings. Consider the previous example, the former concept has associative role ($Disease\_Has\_Metastatic\_Anatomic\_Site$, *Pancreas*), but the later concept has role definition ($Disease\_Excludes\_Metastatic\_Anatomic\_Site$, *Pancreas*). Depending on the inclusion and exclusion of metastatic anatomic locations provided by the role definitions, we can easily distinguish these two concepts.

In this work, to gather as much information as possible, the associative roles we adopt for a concept are the inferred ones that include associative roles inherited from the concept’s ancestors. For instance, in Fig. 2, concept “Cutaneous Pseudolymphoma” contains 18 associative roles (14 inherited from its ancestors), while concept “Non-Neoplastic Skin Disorder” contains three associative roles (two inherited from its ancestors).

### Identifying potentially missing IS-A relations

As mentioned earlier, in this work, our task is to identify potentially missing IS-A relations among candidate pairs—pairs of concepts that are not linked by IS-A relations within non-lattice subgraphs. For each candidate pair (*A*, *B*), we perform a two-step subsumption checking to see if the meaning represented by the hybrid model of *A* is more detailed than *B*’s (i.e., *A* IS-A *B*), or vice versa (i.e., *B* IS-A *A*).

In the first step, we perform a lexical-feature-based checking. We consider original lexical features (i.e., $C_{bow}$, $C_r$) as minimal satisfying features for a concept. In other words, if *A*’s enriched lexical features (i.e., all meanings from lexical features that hold for *A*) satisfy *B*’s original lexical features, we consider *A* is more detailed than *B* in terms of lexical features. Here, we do not consider enriched lexical features of *B* because *A* can then also inherit lexical features from *B*’s ancestors if *A* becomes a subtype of *B*. As we represent lexical features of concepts as sets of words, we simply use set inclusion testing, that is, if *A*’s enriched bag-of-words (i.e., $A_{ebow}$) is a superset of *B*’s bag-of-words (i.e., $B_{bow}$) and *A*’s enriched roots of noun chunks (i.e., $A_{er}$) is a superset of *B*’s roots of noun chunks (i.e., $B_r$), then *A* is considered more detailed than *B* in lexical feature wise.

In the second step, we perform a role-based checking. To do so, we require that each of the two concepts within a candidate pair should contain at least one associative role and associative roles of two concepts should not be totally identical (otherwise we can not decide which one is more detailed). Further, we check that for each associative role ($role_B$, $value_B$) of *B*, if there exists a corresponding role ($role_A$, $value_A$) of *A* such that $role_A$ and $role_B$ are the same and $value_B$ is the same or more general than $value_A$ (i.e.,$value_B$ is an ancestor of $value_A$). If this is the case, then *A* is considered more detailed than *B* in terms of role definitions.

If *A* is more detailed than *B* in terms of both lexical features and role definitions, we consider “*A* IS-A *B*” as a potentially missing IS-A relation. For example, consider a candidate pair (“Cutaneous Pseudolymphoma”, “Non-Neoplastic Skin Disorder”) in Fig. 2. “Cutaneous Pseudolymphoma” is more detailed than “Non-Neoplastic Skin Disorder” in terms of lexical features because the enriched bag-of-words of “Cutaneous Pseudolymphoma”, {cutaneous, pseudolymphoma, benign, lymphoproliferative, disorder, skin, non-neoplastic}, is a superset of bag-of-words of “Non-Neoplastic Skin Disorder”, {non-neoplastic, skin, disorder}; and the enriched roots of noun chunks of “Cutaneous Pseudolymphoma”, {pseudolymphoma, disorder}, is also a superset of roots of noun chunks of “Non-Neoplastic Skin Disorder”, {disorder}. In addition, “Cutaneous Pseudolymphoma” is more detailed than “Non-Neoplastic Skin Disorder” in role definitions, since for each associative role of “Non-Neoplastic Skin Disorder”, there is a corresponding role of “Cutaneous Pseudolymphoma” that is equivalent or more detailed. Therefore, our approach suggests “Cutaneous Pseudolymphoma” IS-A “Non-Neoplastic Skin Disorder” as a potentially missing IS-A relation. Note that this missing IS-A relation has been confirmed by experts from NCI Enterprise Vocabulary Service (EVS) and included in the newer versions of the NCI Thesaurus.

In some cases, a potentially missing IS-A relation detected may be a relation similar to an IS-A relation, such as “$part\ of$”. NCI Thesaurus also provides associations between concepts that are different from role definitions, such as “$Has\_Salt\_Form$”, “$Has\_Target$”, “$Has\_Pharmaceutical\_Transformation$”, etc. We further utilize them to distinguish those IS-A-like relations. Given a potentially missing IS-A relation identified by our approach, if two concepts are already linked by any kind of these associations, then the missing IS-A relation will be abandoned.

Another thing to consider is that due to the large size of some non-lattice subgraphs, there may exist overlaps between non-lattice subgraphs, which may result in redundant missing IS-A relations being suggested. For example, our approach may suggest “*A* IS-A *B*” in one non-lattice subgraph and suggest “*A* IS-A *C*” in another, while *B* is an ancestor of *C* in the current ontology (i.e., an existing IS-A relation). In this case, “*A* IS-A *B*” is considered redundant because it can be implied by the potentially missing IS-A relation“*A* IS-A *C*” and the existing relation “*C* IS-A *B*”. To improve the evaluation efficiency, we avoid unnecessary analyses on such redundant relations. More formally, a detected potentially missing IS-A relation “*A* IS-A *B*” is considered as redundant if it can be inferred by other missing or existing IS-A relations.

## Results

We applied our enhanced hybrid approach to the NCI Thesaurus (19.08d inferred version [25]) for identifying potentially missing IS-A relations.

### Non-lattice subgraphs and suggested IS-A relations

In total, 10,216 non-lattice subgraphs were obtained in 16 sub-hierarchies of the NCI Thesaurus. 55 non-redundant missing IS-A relations were suggested for five sub-hierarchies. Table 2 shows the number of suggested missing IS-A relations for each of the five sub-hierarchies. For example, 34 non-redundant missing IS-A relations were suggested in the “Disease, Disorder or Finding” sub-hierarchy.

**Table 2:** The number of potentially missing IS-A relations identified for sub-hierarchies

Sub-hierarchy	# of Non-lattice subgraphs	# of suggested missing IS-A relations
Disease, disorder or finding	8075	34
Experimental organism diagnosis	257	18
Drug, food, chemical or biomedical material	922	1
Molecular abnormality	143	1
Activity	109	1

### Evaluation

For evaluation, we provided the NCI EVS domain experts, who manage the NCI Thesaurus, with 55 potentially missing IS-A relations identified by our approach. 29 out of 55 were confirmed by EVS experts and have been incorporated in the newer version of the NCI Thesaurus. Table 3 lists ten examples of valid missing IS-A relations verified by EVS experts, including “Glycine Encephalopathy” IS-A “Congenital Nervous System Disorder” and “Congenital Vena Cava Abnormality” IS-A “Congenital Cardiovascular Abnormality”. The detailed evaluation results can be found in Additional file 1: Appendix 1.

**Table 3:** Ten examples of valid missing IS-A relations confirmed by EVS experts

Subconcept	Superconcept
Glycine encephalopathy	Congenital nervous system disorder
Tumor infiltrating lymphocytes-N2-transduced	Therapeutic tumor infiltrating lymphocytes
Stage 0 anal cancer AJCC v8	Anal precancerous condition
Cutaneous pseudolymphoma	Non-neoplastic skin disorder
Congenital vena cava abnormality	Congenital cardiovascular abnormality
Mouse cardiac fibrosarcoma	Mouse cardiac sarcoma
Fibrosarcoma of the mouse intestinal tract	Mouse malignant mesenchymal neoplasm
Carcinoma of the mouse larynx	Mouse carcinoma
Eyelid xanthoma	Non-neoplastic eyelid disorder
Autoimmune lymphoproliferative syndrome-associated lymphoma	Immunodeficiency-related malignant neoplasm

## Discussion

In this work, we combined role definitions and lexical features to suggest missing IS-A relations in the NCI Thesaurus. The evaluation results showed that our hybrid approach is promising in identifying missing IS-A relations. From the true positives, such as “Glycine Encephalopathy” IS-A “Congenital Nervous System Disorder” and “Cutaneous Pseudolymphoma” IS-A “Non-Neoplastic Skin Disorder”, we found that using enriched lexical features for subconcepts helps recognize meanings related to the concepts that cannot be caught from their own concept names.

### Analysis of false positives

Even though our approach correctly suggested missing IS-A relations in the majority of the cases (i.e., 29 out of 55), there were still cases where the approach made incorrect suggestions. By reviewing such invalid IS-A relation suggestions, we identified two major causes for them. The detailed causes for all the false positives are included in Additional file 2: Appendix 2.

The first cause is that the existence of erroneous IS-A relations in the NCI Thesaurus has led to invalid missing IS-A suggestions. For example, our approach suggested “Carcinosarcoma of the Mouse Prostate Gland” IS-A “Carcinoma of the Mouse Prostate Gland” mainly based on an existing IS-A relation “Carcinosarcoma of the Mouse Prostate Gland” IS-A “Mouse Carcinoma”. However, as stated by EVS experts, “carcinosarcoma” is not a kind of “carcinoma”. Thus, the existing IS-A relation on which we rely to derive the missing IS-A relation is incorrect, and it has been fixed by EVS experts in the newer release of the NCI Thesaurus. In total, 7 out of 26 false positive cases fell into this cause. In such cases, even though our suggestions of missing IS-A relations were incorrect, they further revealed problems within the existing hierarchy of the NCI thesaurus that in turn helped improve the quality of the NCI thesaurus.

Secondly, since we only adopted original lexical features ($C_{bow}$, $C_r$) for superconcepts during subsumption testing, the meanings beyond the original lexical features and logical definitions may cause incorrect missing IS-A relations to be suggested. Consider the false positive “Diffuse Pulmonary Lymphangiomatosis” IS-A “Pulmonary Vascular Disorder”. The subconcept is a kind of “neoplasm”, however, the superconcept has an ancestor “Non-Neoplastic Lung Disorder”. Since a neoplasm cannot be a subtype of a non-neoplastic disorder, this suggestion is invalid. Other similar cases include: “Conjunctival Kaposi Sarcoma” IS-A “Conjunctival Vascular Disorder” and “Retinal Hemangioma” IS-A “Retinal Vascular Disorder”. Since meanings like “non-neoplastic” can be found in the enriched lexical features of superconcepts (i.e., inherited from ancestors), a natural question would be: Whether adopting enriched lexical features for both concepts within candidate pairs during lexical-based subsumption testing can improve the performance of our method?

To study this, we further utilized enriched lexical features of superconcept and subconcepts in lexical-based subsumption checking. Therefore, in order for an IS-A relation to be suggested, the enriched lexical features of the subconcept now should also contain the original lexical features of the superconcept’s ancestors. In total, 45 missing IS-A relations were identified in this setting. The result was found to be a subset of our previous result. One exception is that a missing IS-A relation was considered redundant but became non-redundant as some missing IS-A relations are no longer included in the result. Since the IS-A relation was redundant to a valid IS-A relation, this IS-A relation is also considered as valid. Among those 45 missing IS-A relations, 29 were valid IS-A relations, and the number of true positives went down by 3 but the number of false positives went down by 7. We noticed that some false positives in the format of “neoplasm” IS-A “non-neoplastic” still appeared in the result because the role definitions of the superconcepts are not sufficient (i.e., incompleteness). For example, “Kidney Lymphangioma” IS-A “Kidney Vascular Disorder”. The superconcept “Kidney Vascular Disorder” should be a “non-neoplastic” disorder, however, in the role definition, none of its ancestors is “non-neoplastic” disorder and none of its associative roles indicates that it is not a kind of “neoplasm”. Another example is “Brain Astrocytoma” IS-A “Brain Disorder”. Therefore, adopting enriched lexical features for both superconcept and subconcept during lexical-based subsumption checking can improve the performance, but only slightly due to the incompleteness of role definitions.

### Comparison with other approaches

Existing approaches for auditing IS-A relations can be roughly classified into the following categories: structural-based, lexical-based, structural-lexical-based, and machine learning-based. Our hybrid approach in this work falls into the category of structural-lexical-based.

Structural-based approaches utilize the relations between concepts. For instance, abstraction networks (AbNs) [8, 26, 27], which group concepts based on shared outgoing attribute relationships, have been extensively studied to identify problematic areas in ontologies that may contain quality issues, including missing IS-A relations. However, manual review of problematic areas by domain experts are needed to locate and uncover the exact quality issues. Our approach in this work not only identifies problematic areas (i.e., non-lattice subgraphs), but also automatically suggests missing IS-A relations (the actual quality issues) in the problematic areas.

Lexical-based approaches leverage lexical patterns or features of concepts to identify missing IS-A relations. Quesada-Martínez et al. analyzed concept names in the SNOMED CT to identify lexical regularities (LR) and suggest missing relations (including missing IS-A relations) [11]. However, only a small amount of LR can be used to generate missing relations. Bodenreider considered lexical features of concept names as logical definitions of concepts to detect missing IS-A relations [10]. This approach was only applied to two sub-hierarchies of SNOMED CT rooted with concepts “Disorder of head (disorder)” and “Operative procedure on head (procedure)”, respectively. In addition, individual words may not be sufficient to represent the semantic meaning of a concept. In contrast, our approach in this work has been applied to the entire hierarchy of the ontology, and our hybrid model leverages both lexical features and associative roles to represent concepts.

Structural-lexical-based approaches utilize both concepts’ lexical features and relations between concepts. For instance, we have investigated approaches combining non-lattice subgraphs (structural) and lexical features of concepts to automatically detect missing IS-A relations in the SNOMED CT [17, 18] and NCI Thesaurus [19, 20]. The basic idea is first identifying problematic areas (i.e., non-lattice subgraphs) and then leveraging lexical features of concepts to detect missing IS-A relations. In this work, we not only use lexical features but also role definitions of concepts to represent concepts.

The above-mentioned approaches (including our approach in this work) are rule-based. Recently, machine learning-based approaches have been explored to help in detecting missing IS-A relations in biomedical ontologies [13, 21]. Liu et al. used Doc2Vec to learn vector representations for concepts (or concept embeddings) in the NCI Thesaurus, and trained a Convolutional Neural Network (CNN) model facilitated by AbNs to predict if there is an IS-A relation between two given concepts [13]. Sun et al. used Word2Vec to produce word embeddings and aggregated them to obtain concept embeddings for SNOMED CT. A hybrid CNN and multilayer perceptron (CNN-MLP) classifier were developed to predict IS-A relations for given concept pairs. Such machine learning-based approaches highly rely on the strategy of selecting positive/negative samples for training. Although good performance have been achieved for IS-A relation prediction on the pre-constructed training and testing data in the traditional machine learning manner, the trained model cannot be directly usable to uncover missing IS-A relations due to many false predictions, and always need extra assistance (e.g., AbNs in [13] and non-lattice subgraphs in [21]) of providing candidate pairs of concepts to reduce false predictions. In addition, the trained models did not outperform the results of the baseline rule-based approaches. Further work is still needed to improve the performance of machine learning-based approaches.

### Comparison with our previous work

In our previous work [28], we developed a lexical-based approach to identify missing IS-A relations in the NCI Thesaurus. The lexical feature used in that work was the enriched bag-of-words (i.e., $C_{ebow}$ in this paper). Since only one kind of lexical features was used, several other restrictions such as stop words, antonym pairs and location restrictions were applied to avoid potential false identification of missing IS-A relations. In total, 925 potentially missing IS-A relations were identified from 9,512 non-lattice subgraphs in 19.01d inferred version of the NCI Thesaurus. We provided EVS experts with 253 potentially missing IS-A relations in non-lattice subgraphs of size less than or equal to 15. EVS experts confirmed 73 out of 253 suggested missing IS-A relations. We compared our hybrid approach in this paper with the lexical-based approach in previous work [28] in two aspects.

First, we applied our hybrid approach in this work to the 19.01d inferred version of the NCI Thesaurus. In total, 87 non-redundant missing IS-A relations were identified, 56 out of which were obtained from non-lattice subgraphs of size less than or equal to 15. Compared with previously evaluated 253 missing IS-A relations, 28 out of 55 were overlapping. Among those 28 overlapped ones, 14 of them were true positives. Based on this, the precision was improved while the recall was lowered.

In our previous work [28], only one type of lexical features (enriched bag-of-words) was used. Therefore, in the second experiment, we tried to consider associative roles and roots of noun chunks as additional subsumption testing (i.e., in addition to lexical features and other restrictions used in [28]) to further check their effectiveness in helping identify missing IS-A relations. Given 253 missing IS-A relations identified in our previous work, 135 out of 253 were the cases in which both the subconcept and the superconcept contained at least one associative role and their associative roles were not identical. 32 out of 253 cases satisfied the role-based testing, and 14 out of them were valid ones. When it comes to using roots of noun chunks, 245 cases passed the testing, where 70 out of them were valid ones. When it comes to performing additional subsumption testing based on both features, 31 out of 253 passed the testing, where 14 out of them were valid ones. The results indicate that associative roles can be used as the main tester to recognize differences in the intended meanings of concepts and roots of noun chunks can be used to catch the subtle differences.

### Limitations and future work

Although the results showed that our hybrid approach is promising in identifying missing IS-A relations, there are several aspects that could be further improved.

Since we rely on the existing knowledge (including concept names, IS-A hierarchy, and associative roles) of an ontology to detect missing IS-A relations, a limitation of our approach is that the quality issues of the ontology (e.g., incorrect IS-A relations, missing IS-A relations, insufficient associative roles, and incorrect associative roles) may lead to incorrect suggestions of missing IS-A relations in the result. However, manually reviewing how such incorrect missing IS-A relations were obtained by our approach may further help reveal the related quality issues (see subsection “Analysis of False Positives”). Given that such manual review is labor-intensive, it is desirable to further develop systematic ways to help uncover those quality issues.

In this work, we directly used words in concepts names to form lexical features $C_{bow}$ and $C_{ebow}$. Variants of the words (e.g., “disorder” and “disorders”) that may be used to express the same meaning were not considered. As a result, some missing IS-A relations may not be detected. Future work would be to perform normalization by stemming for concept names to identify more missing IS-A relations.

In addition, missing IS-A relations identified by our approach were from 184 out of 10,216 non-lattice subgraphs. New approaches are needed to further reveal potential quality issues in the remaining larger portion of non-lattice subgraphs in the NCI Thesaurus. As mentioned earlier, recent studies [13, 21] have explored the feasibility of machine learning-based approaches to facilitate the identification of missing IS-A relations in biomedical ontologies, indicating that further enhancement is still needed. In future work, we plan to explore how to adapt our hybrid model into embeddings for concepts and leverage machine learning techniques to uncover additional missing IS-A relations.

## Conclusions

In this paper, we introduced a hybrid model that combines lexical features and role definitions of concepts to identify missing IS-A relations within non-lattice subgraphs in the NCI Thesaurus. The results showed that our approach is capable of uncovering valid missing IS-A relations. Further examination of false positives revealed erroneous existing IS-A relations as well as incomplete concept definitions, which in turn also helped improve the quality of the NCI thesaurus. Comparison with our previous lexical-based work further showed the usefulness of leveraging role definitions.

## Supplementary information

**Additional file 1.** Evaluation results for 55 potentially missing IS-A relations identified by our method.

**Additional file 2.**. Causes for false positives.

## Data Availability

The algorithm for detecting potential missing IS-A relations, as well as the results generated and evaluated are available at https://github.com/fengbozheng/BMC_HybridModel.

## Abbreviations

NCI: National Cancer Institute
UMLS: Unified Medical Language System
EVS: Enterprise Vocabulary Service
NLP: Natural Language Processing
LR: Lexical Regularities
